# Effects of dual plasma molecular adsorption system on liver function, electrolytes, inflammation, and immunity in patients with chronic severe hepatitis

**DOI:** 10.1002/jcla.22926

**Published:** 2019-06-17

**Authors:** Gao Chen, Mengzheng Wu, Bibo Wu, Feifei Liu, Jianying Liu, Li Liu

**Affiliations:** ^1^ Department of Infection Deyang People's Hospital Deyang China

**Keywords:** chronic severe hepatitis, dual plasma molecular adsorption system, electrolytes, immune function, liver function

## Abstract

**Background:**

To investigate the effects of dual plasma molecular adsorption system (DPMAS) on the liver function, electrolytes, inflammation, and immunity in patients with chronic severe hepatitis (CSH).

**Methods:**

Total of 162 patients with CSH treated in our hospital from March 2016 to December 2018 were enrolled and equally randomly divided into control group (n = 81) and observation group (n = 81). The patients in control group were treated with plasma exchange, while those in observation group were additionally treated with DPMAS based on the treatment in control group. The liver function, electrolytes, inflammation, and immunity were evaluated and compared between the two groups.

**Results:**

After treatment, the liver function indexes in observation group were significantly favorable compared with those in control group, with the reduction in TBIL, DBIL, ALT, and rise of CHE levels (*P* < 0.05). The levels of K^+^, Na^+^, Cl^−^, and Ca^2+^ in both groups were significantly improved after treatment (*P* < 0.05), although there were no significant differences between the two groups (*P* > 0.05). The levels of C‐reactive protein (CRP), interleukin‐6 (IL‐6), and tumor necrosis factor‐α (TNF‐α) in both groups declined after treatment compared with those before treatment, and those levels in observation group were higher than that in control group (*P* < 0.05). After treatment, the levels of cluster of differentiation 3^+^ (CD3^+^), CD4^+^, and CD4^+^/CD8^+^ were higher in observation group than those in control group, with decreasing level of CD8^+^ (*P* < 0.05).

**Conclusion:**

Dual plasma molecular adsorption system can effectively improve the liver function, effectively correct the electrolyte disorders, reduce the inflammatory response, and adjust the immunity in patients with CSH.

## 1 INTRODUCTION

Chronic severe hepatitis (CSH) is a common digestive system disease in clinic, which is pathologically characterized by the necrosis of a large number of hepatocytes. It even causes death if no timely and effective treatment is adopted.^1^ CSH is difficult to be completely cured, and it frequently relapses easily, causing great physical and emotional pain and seriously affecting the quality of life of patients.^2^ At present, patients with CSH are mainly treated with conventional medical comprehensive treatment, including liver protection therapy and antiviral medication. The therapeutic drugs mainly include adefovir dipivoxil, lamivudine, and entecavir, but the long‐term effects are still not very satisfactory. Besides, the drug toxicity impairs the recovery of liver function and leads to electrolyte imbalance in patients.^3^, ^4^ Plasma exchange is a non‐biological technique through temporarily or partially replacing the liver function, which is effective in the treatment of CSH.^5^ However, simple plasma exchange has certain limitations and poses a potential risk of autoimmune dysfunction. The dual plasma molecular adsorption system (DPMAS) is a typical non‐biological artificial liver technique that improves the efficacy of simple plasma exchange.^6^ In the present study, we evaluate the effect of simple plasma exchange combined with DPMAS for the treatment of patients with CSH, so as to provide a basis for the development of artificial liver treatment of CSH.

## 2 DATA AND METHODS

### 2.1 General data

A total of 162 patients with CSH treated in our hospital from March 2016 to December 2018 were enrolled and equally randomly divided into control group (n = 81) and observation group (n = 81) using a random number table. Inclusion criteria were as follows: (a) patients meeting the diagnostic criteria for CSH,^7^ (b) patients treated with medical comprehensive treatment, and (c) patients who or whose families signed the informed consent. Exclusion criteria were as follows: (a) patients complicated with severe infection or severe cardiovascular and cerebrovascular diseases or mental diseases, (b) patients allergic to blood products or drugs used in treatment, or (c) pregnant or lactating women. There were no statistically significant differences in the general data between the two groups (*P* > 0.05) (Table 1).

**Table 1:** General data of objects of study

Item	Control group (n = 81)	Observation group (n = 81)	*t*/*χ* ^2^	*P*
Gender (male/female)	43/38	46/35	0.224	0.636
Age (years old)	31‐65	30‐69		
Mean age (years old)	46.13 ± 5.57	45.83 ± 5.72	0.338	0.736
BMI (Kg/m^2^)	21.74 ± 1.37	21.61 ± 1.63	0.550	0.583
Duration of disease (mo)	5.82 ± 3.35	5.74 ± 3.57	0.147	0.883
Type of hepatitis (n [%])				
Hepatitis B	60 (74.07)	64 (79.01)	0.550	0.458
Hepatitis C	21 (25.93)	16 (19.75)	0.876	0.349

### 2.2 Methods

#### 2.2.1 Treatment methods

The patients in both groups were treated with medical comprehensive treatment, including intravenous infusion of compound glycyrrhizin, other liver protection treatment, oral administration of entecavir tablets for antiviral therapy, and bed rest. Based on the above treatment, the patients in control group were treated with simple plasma exchange: Gambro AK100 (GAMBRO LUNDIA MONITOR DIVISION) plasma exchange machine and D‐type (Braun) hemodialysis machine were used. Low molecular heparin (50 U/kg) was used for anticoagulation, 2500 mL plasma was separated and discarded on average using a fractional plasma separator, and 60 g 10% albumin + 2500 mL Riger's mixture were intravenously injected at a rate of 10 mL/min. The blood was exchanged (160 mL/min) for about 2 hours and the changes in such vital signs as pulse. The blood pressure and respiration were closely observed during the plasma exchange. The patients in observation group received the treatment of DPMAS combined with plasma exchange: After the plasma was separated, it was delivered to a disposable plasma bilirubin adsorber and neutral macroporous resin hemoperfusion apparatus for dual adsorption. Then, patients received the treatment for 3 hours at a flow rate of 120 mL/min. The patients in both groups were treated once or twice a week Artificial liver support system is CRRT (B.Braun Germany).

#### 2.2.2 Detection of indexes

After 5 mL venous blood was drawn from patients (fasting for 8 hours) before and after treatment, it was centrifuged (Hunan Changsha Xiangrui Centrifuge Co., Ltd.) at 600 *g* for 30 minutes. The supernatant was taken and stored in a refrigerator at −20°C. The levels of cluster of differentiation 3^+^ (CD3^+^), CD4^+^, and CD8^+ ^were detected using a flow cytometer (Partec). The levels of serum interleukin‐6 (IL‐6), tumor necrosis factor‐α (TNF‐α), and C‐reactive protein (CRP) were detected via enzyme‐linked immunosorbent assay (ELISA) strictly according to the instructions of kits (Thermo Fisher Scientific). The optical density (OD) value was read at a wavelength of 450 nm using a microplate reader, and the concentrations of IL‐6, TNF‐α, and CRP were calculated. The liver function was detected after treatment using a full‐automatic biochemical analyzer (Toshiba), and the relevant indexes were serum total bilirubin (TBIL), direct bilirubin (DBIL), alanine aminotransferase (ALT), and cholinesterase (CHE). The levels of serum electrolytes, including K^+^, Na^+^, Cl^−^, and Ca^2+^, were analyzed using aDSI‐905 full‐automatic electrolyte analyzer (Shanghai Xunda Medical Instrument Co., Ltd.) before and after treatment, and the concentration of each ion was calculated using a tester based on the standard curve.

### 2.3 Evaluation criteria

The levels of serum TBIL, DBIL, ALT, and CHE were measured and compared between the two groups after treatment. The electrolyte levels in both groups were detected before and after treatment, including K^+^, Na^+^, Cl^−^, and Ca^2+^. The levels of serum inflammatory factors (CRP, IL‐6, and TNF‐α) were analyzed in both groups via ELISA before treatment and at 1, 3, and 6 months after treatment. The levels of T lymphocyte subsets (CD3^+^, CD4^+^, CD8^+^, and CD4^+^/CD8^+^) were determined via flow cytometry after treatment.

### 2.4 Statistical processing

SPSS 19.0 (SPSS Inc) software was used for data processing. Measurement data were expressed as mean ± standard deviation ($\overset{¯}{x}\pm s$). *t* Test was used for comparison between two groups, and chi‐square test was used for enumeration data. Continuous data from multiple groups were analyzed by using one‐way ANOVA, with the Tukey post hoc test. *P* < 0.05 suggested that the difference was statistically significant.

## 3 RESULTS

### 3.1 Comparison of liver function between the two groups of patients

After treatment, the levels of TBIL, DBIL, and ALT in observation group were significantly lower than those in control group, while the level of CHE was significantly higher than that in control group (*P* < 0.05) (Table 2).

**Table 2:** Comparison of liver function between the two groups of patients

Group	n	TBIL (μmol/L)	DBIL (μmol/L)	ALT (U/L)	CHE (U/L)
Observation group	81	184.21 ± 16.79	91.39 ± 4.28	142.57 ± 13.69	1237.02 ± 438.63
Control group	81	294.51 ± 36.42	125.45 ± 5.69	171.39 ± 18.65	5702.47 ± 362.88
*t*		24.75	43.05	11.21	70.60
*P*		<0.001	<0.001	<0.001	<0.001

### 3.2 Comparisons of electrolyte levels between the two groups of patients

The electrolyte levels, including K^+^, Na^+^, Cl^−^, and Ca^2+^, in both groups were significantly improved after treatment (*P* < 0.05), but there were no significant differences between the two groups (*P* > 0.05) (Tables 3 and 4).

**Table 3:** K^+^ and Na^+^ levels before and after treatment in both groups of patients

Group	K^+^ (mmol/L)				Na^+^ (mmol/L)			
	Before treatment	After treatment	*t*	*P*	Before treatment	After treatment	*t*	*P*
Observation group	3.63 ± 0.41	4.53 ± 0.64	10.660	<0.001	134.72 ± 3.81	139.27 ± 3.85	7.560	<0.001
Control group	3.61 ± 0.57	4.83 ± 0.61	11.530	<0.001	135.31 ± 3.70	138.37 ± 3.66	5.335	<0.001
*t*	0.256	3.054			1	1.537		
*P*	0.798	<0.01			0.319	0.126		

**Table 4:** Cl^−^ and Ca^2+^ levels before and after treatment in both groups of patients

Group	Cl^−^ (mmol/L)				Ca^2+^ (mmol/L)			
	Before treatment	After treatment	*t*	*P*	Before treatment	After treatment	*t*	*P*
Observation group	96.80 ± 1.59	97.52 ± 2.06	2.490	0.0138	2.17 ± 0.37	2.75 ± 0.46	8.842	<0.001
Control group	96.87 ± 1.78	99.63 ± 1.83	9.73	<0.001	2.14 ± 0.48	2.64 ± 0.38	7.350	<0.001
*t*	0.264	6.892			0.445	1.659		
*P*	0.792	<0.001			0.657	0.100		

### 3.3 Comparisons of inflammatory levels between the two groups of patients before and after treatment

We evaluated the inflammatory response, and we found that the levels of CRP, IL‐6, and TNF‐α in both groups obviously declined after treatment compared with those before treatment. As the treatment time extended, the levels of inflammatory factors significantly decreased in observation group compared with that in control group (*P* < 0.05) (Tables 5, 6 and 7).

**Table 5:** Comparison of CRP level between the two groups of patients before and after treatment (ng/mL)

Group	n	Before treatment	Week 1	Week 2	Week 4	*F*	*P*
Observation group	46	68.49 ± 3.53	48.03 ± 3.89	39.12 ± 3.79	31.57 ± 3.48	1524	<0.0001
Control group	46	68.12 ± 3.63	54.26 ± 3.73	43.82 ± 3.69	37.52 ± 3.72	1063	<0.0001
*t*		0.658	10.400	7.997	10.510		
*P*		0.512	<0.001	<0.001	<0.001		

**Table 6:** Comparison of TNF‐α level between the two groups of patients before and after treatment (pg/mL)

Group	n	Before treatment	Week 1	Week 2	Week 4	*F*	*P*
Observation group	46	47.82 ± 3.70	31.32 ± 3.52	25.62 ± 3.73	19.53 ± 3.83	876.3	<0.0001
Control group	46	47.86 ± 3.74	35.42 ± 3.74	31.53 ± 3.63	27.83 ± 3.95	432.5	<0.0001
*t*		0.068	7.185	10.220	13.580		
*P*		0.946	<0.001	<0.001	<0.001		

**Table 7:** Comparison of IL‐6 level between the two groups of patients before and after treatment (pg/mL)

Group	n	Before treatment	Week 1	Week 2	Week 4	*F*	*P*
Observation group	46	155.89 ± 3.74	127.74 ± 3.82	106.42 ± 3.73	94.63 ± 3.83	4101	<0.001
Control group	46	156.02 ± 3.63	135.73 ± 4.12	123.02 ± 4.34	116.62 ± 3.65	1569	<0.001
*t*		0.225	12.800	26.110	37.410		
*P*		0.823	<0.001	<0.001	<0.001		

### 3.4 Comparison of immunity between the two groups after treatment

After treatment, the levels of CD3^+^, CD4^+^, and CD4^+^/CD8^+^ were higher in observation group than those in control group, while the level of CD8^+^ was lower than that in control group (*P* < 0.05) (Table 8).

**Table 8:** Comparisons of T lymphocyte subsets between the two groups of patients after treatment

Group	n	CD3^+^	CD4^+^	CD8^+^	CD4^+^/CD8^+^
Observation group	46	70.04 ± 3.79	41.35 ± 3.77	28.94 ± 3.74	1.86 ± 0.58
Control group	46	67.47 ± 3.84	38.52 ± 3.82	26.44 ± 3.59	1.57 ± 0.64
*t*		4.287	4.476	4.430	3.022
*P*		<0.001	<0.001	<0.001	0.003

## 4 DISCUSSION

The liver is a vital organ in the human body, which significantly participates in the metabolism, detoxification, excretion, and synthesis functions. In the case of short‐term liver dysfunction under the influence of various adverse factors, CSH will occur.^8^ In patients with CSH, there is massive accumulation of toxic metabolites, leading to severe liver cell damage, hemorrhage, infection, hepatic encephalopathy, and hepatorenal syndrome, which induces hepatic failure and functional decompensation, with the mortality rate up to 50%Revision: It should be 5%, not 50%.^9^, ^10^ The pathogeneses of CSH include metabolic, infectious, drug, and other factors. The cytotoxic substances accumulated in patients with CSH cannot be effectively removed via medical treatment, which impairs the inhibition of the liver cell damage, failing to achieve a satisfactory therapeutic effect.^11^ The artificial support system contributes to a certain therapeutic effect on CSH, which favors the rehabilitation of patients.^12^

A large number of liver cells necrotize in patients with CSH under the influence of various factors. The impairment of liver function induces a series of complications, including the deterioration of the detoxification, synthesis, biotransformation, the immune response, resulting in the accumulation of cytotoxic substances in the body, and in turn further damaging the liver function.^13^ The results of the present study revealed that the liver function was recovered after plasma exchange treatment in both groups. The levels of TBIL, DBIL, and ALT in observation group were remarkably lower than those in control group, while the CHE level was remarkably higher than that in control group (*P* < 0.05). The reason is that the plasma exchange can temporarily replace part of the patient's liver function, thus creating a favorable internal environment for autologous hepatocyte regeneration and repair, and gradually improving the liver function. Plasma exchange combined with DPMAS can enhance the removal of bilirubin and effectively increase the prothrombin activity, thereby raising the liver viability and significantly improving the patient's liver function.^14^

Due to the serious injury of liver function, patients with CSH suffer from nausea and vomiting, loss of appetite, and decline in gastrointestinal absorption, thus the intake of electrolytes, such as K^+^, Na^+^, Cl^−^, and Ca^2+^ is reduced. Moreover, the medical comprehensive treatment will aggravate the electrolyte disorders in the body, leading to electrolyte imbalance.^15^ In the present study, the levels of K^+^, Na^+^, Cl^−^, and Ca^2+^ in both groups were significantly improved after treatment (*P* < 0.05), but basically there were no significant differences between the two groups (*P* > 0.05). We proposed that in both simple plasma exchange and plasma exchange combined with DPMAS, the toxins accumulated in patients are removed via convection, diffusion, and adsorption. The electrolytes required in the body are supplemented, and electrolyte imbalance is obviously improved.

Tumor necrosis factor‐α is one of the most important inflammatory mediators, which can initiate and trigger inflammatory responses and cause multiple signaling cascades.^16^ IL‐6 induces adhesion and aggregation of inflammatory cells to promote inflammation, which plays diverse roles in the body.^17^ CRP represents a commonly used inflammatory index in clinic.^18^ During the process of necrosis of liver cells in patients with CSH, the endotoxin increases, which strongly induces monocyte‐macrophages to secrete a large amount of TNF‐α. The increasing level of TNF‐α can elicit the production of CRP, IL‐6, and other cytokines to be involved in the inflammatory response in the liver, cause secondary damage to liver cells, aggravate liver damage, and deteriorate the disease.^19^ The results of this study showed that the levels of CRP, IL‐6, and TNF‐α in both groups significantly declined after treatment compared with those before treatment, and DPMAS even decreased these levels. It is believed that plasma exchange and DPMAS can drain the blood from patients and separate plasma and cell components using the plasma separator, which can not only effectively scavenge endotoxin, but also efficaciously remove such cytokines as CRP, IL‐6, and TNF‐α. Then, the cell components and fresh plasma in the same type are transfused into patients, thus effectively reducing the levels of inflammatory factors.

The inflammatory response in patients with CSH can lead to immune dysfunction, cause secondary immune damage, up‐regulate the expression of CD8^+^ T lymphocytes, and initiate apoptosis.^20^ We found that after treatment, the levels of CD3^+^, CD4^+^, and CD4^+^/CD8^+^ in observation group were higher than those in control group, while the level of CD8^+^ was lower than that in control group. DPMAS is considered to increase the effect of simple plasma exchange, and such combined application reduces the loss of beneficial substances in the patient's plasma, such as blood coagulation factors, albumin, and growth factors, therefore attenuating the damage of platelets and erythrocytes, and enhancing the immunity in patients.

## 5 CONCLUSION

In conclusion, plasma exchange combined with DPMAS can effectively improve the liver function, regulate the electrolyte balance, reduce the inflammatory response, and enhance the immunity in patients with CSH. It serves as an enhancer to simple plasma exchange and benefits the clinical popularization and application.
